# A case of malignant glomus tumor (glomangiosarcoma) of the nasal cavity

**DOI:** 10.1093/jscr/rjab641

**Published:** 2022-01-26

**Authors:** Omar A Alhroub, Shimaa A Mahameed, Mohammad O Abdelhafez, Asil Alhroub, Hani Hour, Nabil Hasasna, Nazmi Kamal

**Keywords:** nasal cavity, glomus tumor, glomangiosarcoma

## Abstract

Glomus tumors are rare and usually benign. The malignant form (glomangiosarcoma) comprises <1% of all glomus tumors. There are limited reports that describe glomus tumors in the nasal cavity. However, to the best of our knowledge, glomangiosarcoma of the nasal cavity was never reported in humans. We report on the first case of nasal cavity glomangiosarcoma in a 59-year-old male who presented with a bleeding mass in his right nostril. We completely excised the lesion with a 0.7-mm free margin, and the histopathologic examination was consistent with glomangiosarcoma. A 6-month follow-up illustrated no evidence of recurrence or distant metastasis. Although it is rare, glomus tumors should be in the differential diagnosis of nasal cavity tumors. Histopathologic examination is essential for glomangiosarcoma diagnosis. Treatment requires complete excision with free margin, alongside careful clinical and radiological follow-up.

## INTRODUCTION

Glomus tumor is mesenchymal neoplasm originating from the glomus body, modified arteriovenous shunts involved in thermoregulation; they are rare neoplasms, mostly found in the older population with a higher incidence in females [1, 2]. It usually arises in the distal extremities where glomus bodies are most abundant; however, it can grow anywhere in the body. As a result, it can present with variant localizing signs and symptoms, which makes the diagnosis challenging [3, 4]. The malignant variant (glomangiosarcoma) is exceptionally uncommon, accounting for <1% of all glomus tumors [5]. There are limited reported cases of benign glomus tumors (glomangioma) arising in the nasal cavity [6]. However, a glomangiosarcoma of the nasal cavity has never been described in the literature. Here we report on the first case of a malignant glomus tumor in the nasal cavity.

## CASE PRESENTATION

**Figure 1:**
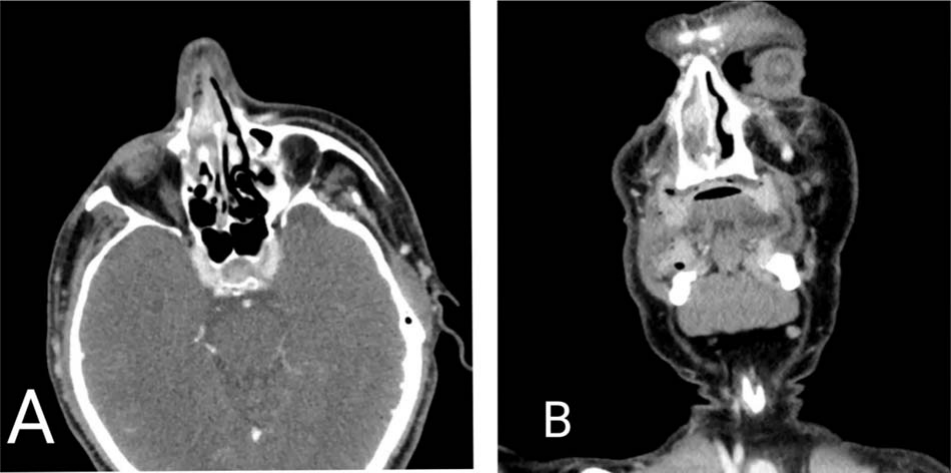
Computed tomography scan of the head. (**A**) Axial view and (**B**) coronal view demonstrating mass lesion in the right nasal cavity attached to the septum measuring 1 × 1.5 cm.

A 59-year-old male with a history of intermittent epistaxis of 20-day duration presented with active bleeding from his right nostril, which was refractory to compression; we applied nasal packing with adrenaline, with only minimal improvement. Physical examination revealed a red-purple mass lesion obstructing the right nasal passage. A computed tomography scan showed a mass in the right nasal cavity attached to the septum measuring 1 × 1.5 cm, shown in Figure 1. Therefore, urgent complete surgical excision through functional endoscopic sinus surgery was done, besides sphenopalatine artery ligation, with no complications.

**Figure 2:**
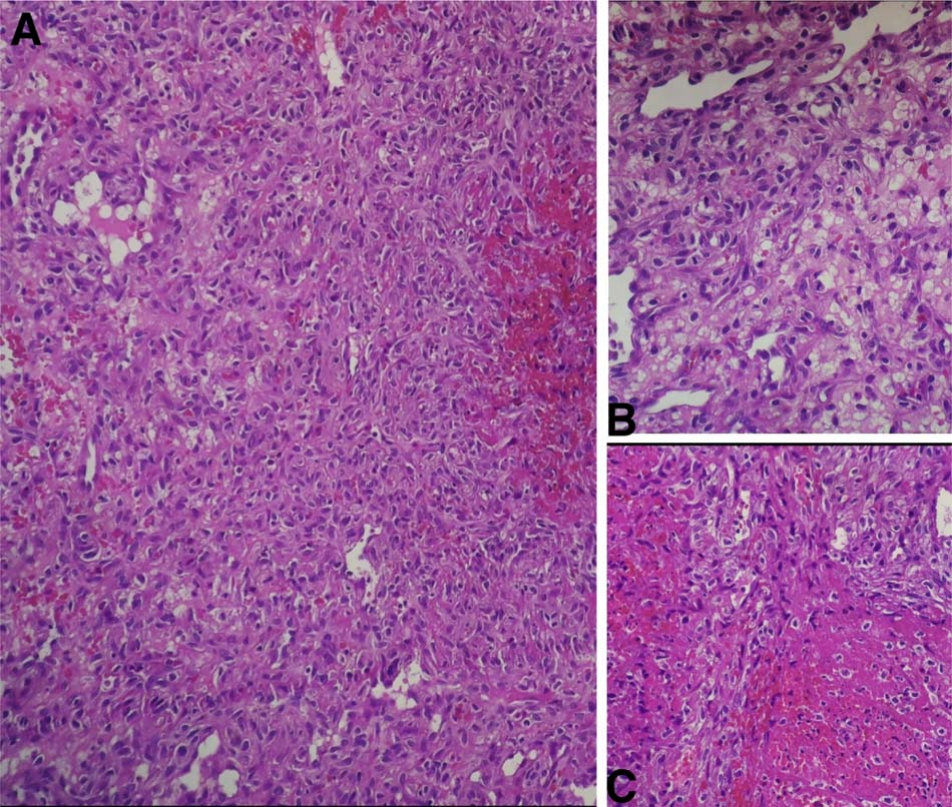
(**A**) Intermediate-power field (×20) shows marked nuclear atypia and pleomorphism. (**B**) High-power field (×40) shows proliferation of atypical spindle cells with atypical mitotic figures (**C**) High-power field (×40) shows focal areas of necrosis.

**Figure 3:**
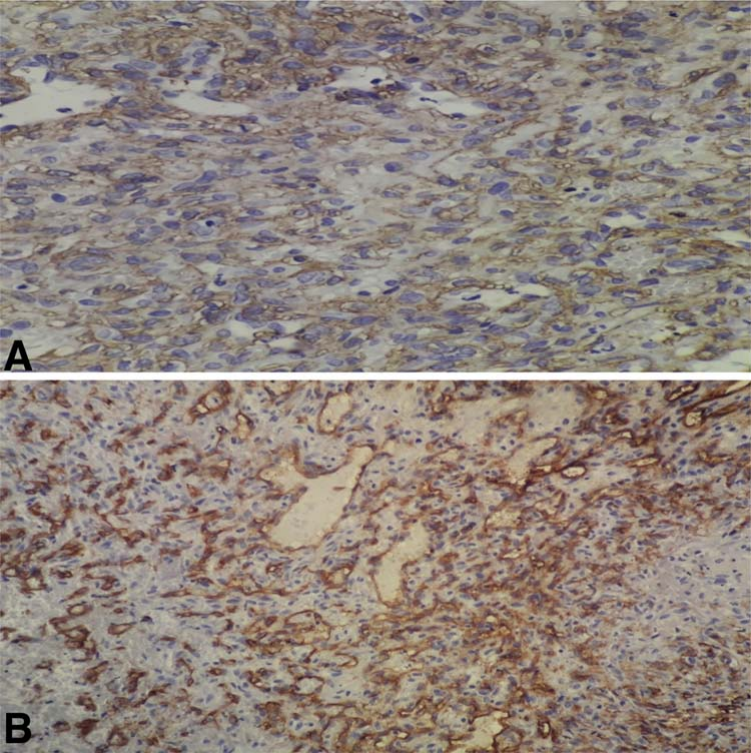
(**A**) High-power field (×40) shows immunoreactivity for SMA; (B) high-power field (×40) shows focal immunoreactivity for CD34.

**Figure 4:**
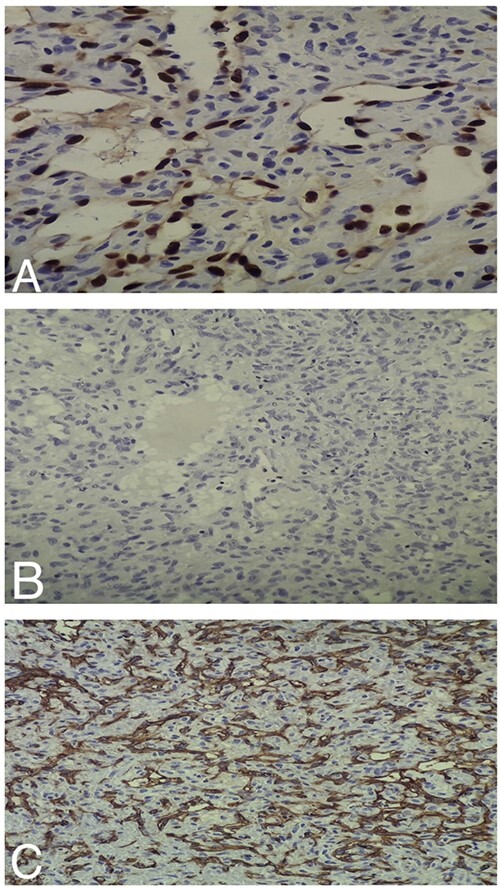
(**A**) Intermediate-power field (×20) shows negative ERG staining by the tumor cells. (**B**) Intermediate-power field (×20) shows negative S100 staining by the tumor cells. (**C**) Intermediate-power field (×20) shows negative CD31 staining by the tumor cells.

Histopathologic examination of the resected mass showed proliferation of atypical spindle cells with atypical mitotic figures, focal areas of necrosis, marked nuclear atypia and pleomorphism (Fig. 2). Immunohistochemical analyses of the tumor cells were positive for smooth muscle actin (SMA) besides focal immunoreactivity for CD34 (Fig. 3). However, ERG, CD31, S100, H-caldesmon, beta-catenin, STAT6 and CKmnf-16 were negative (Fig. 4). Histopathological findings were consistent with malignant glomus tumors.

On postoperative follow-up, the patient had no complications and was improving. He had magnetic resonance imaging 3-week post-surgery, which showed a faint enhancing area of 5 mm seen in the right nasal cavity, likely representing a focal area of post-surgical changes. There was no evidence of any lesions. The patient has had a positron emission tomography (PET) scan 2 weeks later, which demonstrated no signs of distant metastasis. Still, there was active, hypermetabolic soft tissue density in the right nasal cavity; this probably was a benign post-surgical finding. Yet, we could not exclude malignant growth. We performed another surgical intervention through a midface degloving approach including resection of the suspicious lesion along with ~0.7-mm grossly free margin, besides taking biopsies from the margins and resection of the cartilage preserving the mucosa on the other side.

Histopathologic evaluation of the resected lesion revealed no evidence of residual disease and exhibited a picture of chronic inflammation and no signs of malignancy. We performed a PET scan 6 months later, and it showed no signs of active lesions or distant metastasis.

## DISCUSSION

Most glomus tumors are small neoplasms that are benign, usually arise in the subungual zones of the digits where glomus bodies are most abundant, but they still can originate in unusual sites such as deep soft tissues, gastrointestinal tract, respiratory tract and rarely in the head and neck [6]. Glomangiosarcoma is exceedingly rare, composing <1% of all tumors [5]. To the best of our knowledge, glomangiosarcoma in the nasal cavity was never reported in humans. The only reported case of glomangiosarcoma in the nasal cavity was in a horse [7].

Glomus tumors of the nasal cavity mainly manifested with epistaxis and obstructive symptoms. Yet, patients with localized pain and asymptomatic cases were reported [6]. Our patient presented with heavy bleeding from his right nostril with nasal obstruction; the bleeding was refractory to nasal compression. He also had a prior history of intermittent episodes of self-limited epistaxis for 20-day duration.

The clinical symptoms with radiologic findings are usually insufficient to reach a definite diagnosis. However, hematoxylin and eosin staining, coupled with immunohistochemical staining, is required to achieve the definitive diagnosis of glomangiosarcoma [8].

Histologically, the classic features of glomus tumors are uniform sheets of small, round cells with round to oval nuclei [9]. The tumor mainly encompasses three components; glomus cells, vasculature and smooth muscle cells [10]. Glomus tumors typically stain positive for α-SMA, muscle-specific actin, vimentin, calponin, focal CD34, collagen type IV and h-caldesmon. It is almost always negative for CD31, cytokeratin, S100, HMB45 and CD117 [9, 10].

Glomus tumors have a broad differential diagnosis. When they arise in the nasal cavity, we should distinguish them from other tumors, such as hemangiopericytoma and angiosarcoma. The distinction between hemangiopericytoma and glomus tumors is based on the expression of smooth muscles features found in glomus tumors, such as positive staining for SMA, whereas hemangiopericytomas lack these features [11]. Angiosarcoma is a vascular neoplasm that stains strongly positive for the vascular markers CD31 and ERG transcription factor [12, 13]. However, glomus tumors stain negatively for these markers [9, 13], as observed in our patient (Fig. 4).

Folpe *et al*. classified glomus tumors with atypical features into glomangiosarcoma, a malignant glomus tumor; symplastic glomus tumor presents with nuclear atypia alone; glomangiomatosis a glomus tumor with diffuse growth and benign histological features and glomus tumor of uncertain malignant potential.

They suggested considering glomangiosarcoma in lesions that meet at least one of the following criteria, deep-sited location with a size >2-cm, atypical mitotic figures, or the combination of moderate to high nuclear grade and mitotic activity (5 mitoses/50 high-power field (HPF)). They found the metastasis rate in glomangiosarcoma cases can be as high as 40% [5]. Therefore, close clinical and radiological follow-up is mandatory for these patients.

Our patient’s tumor was consistent with Folpe *et al*., criteria for diagnosing glomangiosarcoma as the tumor displayed atypical mitotic figures; these, along with the presence of focal necrosis, nuclear atypia and pleomorphism, are alarming features for high metastatic risk.

We approached the mass initially by complete excision followed by histopathologic evaluation. Then, we resected the remnant hypermetabolic lesion with a 0.7-mm free margin without adjuvant chemotherapy or radiotherapy, besides close clinical and radiologic follow-up. The patient had no local recurrence or distant metastasis after a 6-month follow-up. Based on our experience, complete excision of the mass with macroscopic free margins is curative. However, glomangiosarcoma has a fair metastatic and recurrence rate. Therefore, clinical and radiological follow-up for such patients is crucial.

## References

[1] Kilmpasanis A, Apazidi-Kesoglou Z, Poutoglidis A, Sotiroudi S, Vlachtsis K, Tsetsos N. Nasal septum glomus tumor: a rare cause of unilateral nasal obstruction. Ear Nose Throat J. April 2021. 10.1177/01455613211007948. 33829885

[2] Meguro S, Kusama Y, Matsushima S, et al. Nasal glomus tumor: a rare nasal tumor with diffuse and strongly positive synaptophysin expression. Pathol Int 2019;69:672. 31682049 10.1111/pin.12866 PMC6899972

[3] Schiefer TK, Parker WL, Anakwenze OA, Amadio PC, Inwards CY, Spinner RJ. Extradigital glomus tumors: a 20-year experience. Mayo Clin Proc 2006;81:1337–44. 17036559 10.4065/81.10.1337

[4] Ajala RT, Lyon KA, Lyon PR, Harris FS. Extradigital glomus tumor mimics an intrinsic nerve tumor in a trauma patient: case report and literature review. Cureus 2021;13:e19256. 34900456 10.7759/cureus.19256 PMC8648149

[5] Folpe AL, Fanburg-Smith JC, Miettinen M, Weiss SW. Atypical and malignant glomus tumors: analysis of 52 cases, with a proposal for the reclassification of glomus tumors. Am J Surg Pathol 2001;25:1–12. 11145243 10.1097/00000478-200101000-00001

[6] Koh YW, Lee BJ, Cho KJ. Glomus tumor of the sinonasal tract: two case reports and a review of literature. Korean J Pathol 2010;44:326–9.

[7] Peters M, Grafen J, Kuhnen C, Wohlsein P. Malignant glomus tumour (glomangiosarcoma) with additional neuroendocrine differentiation in a horse. J Comp Pathol 2016;154:309–13. 27102445 10.1016/j.jcpa.2016.03.002

[8] Wang Y, Xiang Y, Bian Y, et al. Glomus tumors associated with the bone and joints: a review of 91 cases. Ann Transl Med 2020;8:1460–0. 33313205 10.21037/atm-20-6998 PMC7723542

[9] Mravic M, LaChaud G, Nguyen A, Scott MA, Dry SM, James AW. Clinical and histopathological diagnosis of glomus tumor: an institutional experience of 138 cases. Int J Surg Pathol 2015;23:181–8. 25614464 10.1177/1066896914567330 PMC4498398

[10] Pathology Outlines . Glomus tumor. https://www.pathologyoutlines.com/topic/softtissueglomus.html (1 December 2021, date last accessed).

[11] Porter PL, Bigler SA, McNutt M, Gown AM. The immunophenotype of hemangiopericytomas and glomus tumors, with special reference to muscle protein expression: an immunohistochemical study and review of the literature. Mod Pathol 1991;4:46–52. 1708501

[12] Ohsawa M, Naka N, Tomita Y, Kawamori D, Kanno H, Aozasa K. Use of immunohistochemical procedures in diagnosing angiosarcoma. Evaluation of 98 cases. Cancer 1995;75:2867–74. 7773935 10.1002/1097-0142(19950615)75:12<2867::aid-cncr2820751212>3.0.co;2-8

[13] Miettinen M, Wang ZF, Paetau A, et al. ERG transcription factor as an immunohistochemical marker for vascular endothelial tumors and prostatic carcinoma. Am J Surg Pathol 2011;35:432–41. 21317715 10.1097/PAS.0b013e318206b67b PMC6880747

